# SQSTM1/p62 in intrahepatic cholangiocarcinoma promotes tumor progression via epithelial–mesenchymal transition and mitochondrial function maintenance

**DOI:** 10.1002/cam4.4908

**Published:** 2022-06-08

**Authors:** Jiafeng Chen, Zheng Gao, Xiaogang Li, Yinghong Shi, Zheng Tang, Weiren Liu, Xin Zhang, Ao Huang, Xuanming Luo, Qiang Gao, Guangyu Ding, Kang Song, Jian Zhou, Jia Fan, Xiutao Fu, Zhenbin Ding

**Affiliations:** ^1^ Department of Liver Surgery & Transplantation, Liver Cancer Institute Zhongshan Hospital, Fudan University Shanghai China; ^2^ Key Laboratory of Carcinogenesis and Cancer Invasion Chinese Ministry of Education Shanghai China; ^3^ Shanghai Xuhui Central Hospital Zhongshan‐Xuhui Hospital, Fudan University Shanghai China

**Keywords:** EMT, intrahepatic cholangiocarcinoma, metastasis, mitophagy, SQSTM1/p62

## Abstract

**Background:**

SQSTM1/p62 is a selective autophagy receptor that regulates multiple signaling pathways participating in the initiation and progression of tumors. Metastasis is still the main cause for intrahepatic cholangiocarcinoma (ICC)‐associated mortality. Hence, this study aimed to explore the mechanism of p62 promoting the progression of ICC.

**Methods:**

Western blotting and immunohistochemical analyses were conducted to detect the expression level of protein p62 in ICC tissues and its correlation with prognosis. Subsequently, the loss‐of‐function experiments in vitro and in vivo were performed to define the role of p62 in ICC cell proliferation, invasion, and metastasis. Then, the effect of p62 knockdown on mitochondrial function and mitophagy was evaluated by measuring the oxygen consumption rate, and using immunofluorescence and western blotting analyses.

**Results:**

The expression of p62 was significantly upregulated in ICC specimens compared with normal tissues. We further illustrated that p62 expression positively correlated with lymph node metastasis and poor prognosis. The loss‐of‐function assays revealed that p62 not only promoted ICC cell proliferation, migration, and invasive capacities in vitro, but also induced lung metastasis in the xenograft mouse model. Mechanistically, high expression of p62‐induced epithelial–mesenchymal transition (EMT) with the upregulation of Snail, vimentin, N‐cadherin, and downregulation of E‐cadherin. Moreover, the autophagy‐dependent function of p62 might play a vital role in maintaining the mitochondrial function of ICC by mitophagy which might further promote EMT.

**Conclusion:**

These data provided new evidence for the mechanism by which abundant p62 expression promoted ICC progression, suggesting a promising therapeutic target for antimetastatic strategies in patients with ICC.

## Graphical abstract

SQSTM1/p62 not only promoted ICC cells proliferation, migration and invasive capacity in vitro, but also induced lymph‐node metastasis in vivo by inducing epithelial‐mesenchymal transition process. And the autophagy‐dependent function of p62 may play a vital role in EMT of ICC by mitophagy.

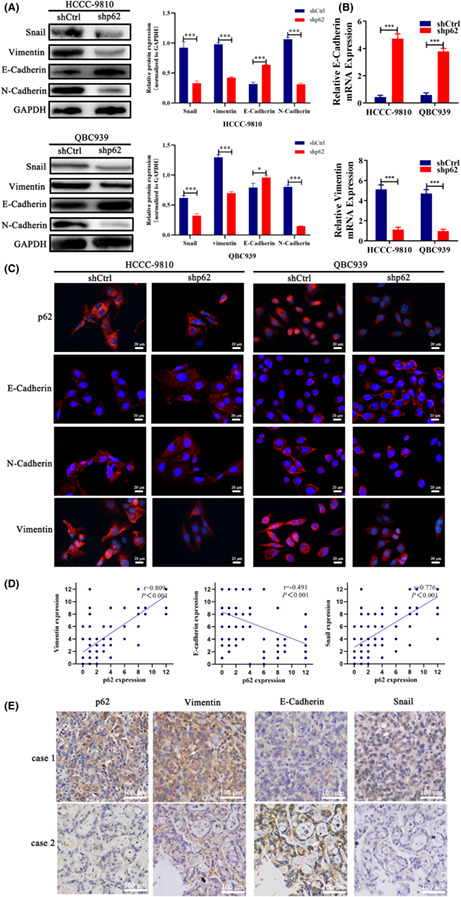

## 1 INTRODUCTION

Multifunctional adaptor protein SQSTM1/p62, the first selective autophagy receptor identified in metazoans, can deliver polyubiquitinated proteins or damaged organelles to autophagosomal‐lysosomal for degradation.^1^ The role of p62 in autophagy has been well investigated, while it is also indicated that p62 acts as an oncoprotein and promotor of tumor progression through multiple signaling pathways.^2^, ^3^, ^4^, ^5^ In the last decades, increasing evidence demonstrated the accumulation of p62 in various tumors, including hepatocellular carcinoma (HCC) and intrahepatic cholangiocarcinoma (ICC).^2^, ^3^, ^4^, ^6^ High p62 expression facilitates the development of HCC via Keap1‐Nrf2, mTORC1, and c‐Myc pathways.^4^ However, the expression and underlying mechanism by which p62 influences ICC progression remains largely unexplored.

Epithelial–mesenchymal transition (EMT) and mitochondrial metabolic reprogramming are two different processes to respond to the stress from tumor microenvironment (TME) which present an intricate correlation in the progression of tumors. Intrahepatic invasion and distal metastasis of ICC need to acquire migratory properties by a cell transdifferentiation process known as EMT. During EMT, the epithelial cells adopt functional and structural characteristics of mesenchymal cells that cause them to lose polarity.^7^ The most important transcription factors that drive EMT include SNAI1, TWIST1, and ZEB1 as well as noncanonical transcription factors such as FOXC2 and GSC.^8^ Mitochondria are central organelles in metabolic reprogramming which contribute to tumor initiation, progression, and drug resistance.^9^ Cancer cells meet the bioenergetic requirements through maintaining mitochondrial function.^10^ However, it also has been reported that mitochondrial dysfunction, which is caused by deregulated mitophagy, depletion of mitochondrial DNA, or mutations in Krebs' cycle enzymes, can promote EMT activation in cancer cells.^11^

Mitochondrial homeostasis plays a vital role in fueling cancer initiation and progression which depends on the balance of degradation and biogenesis. Autophagy serves as an evolutionarily catabolic pathway through “self‐digestion” of cytoplasmic components upon microenvironmental or therapeutic stress to maintain cell homeostasis.^12^ Besides macroautophagy, mitophagy, which acts as the quality control of mitochondrial function, is one form of selective autophagy that specifically recognizes and degrades damaged mitochondria mediated by autophagy receptors, such as OPTN, NBR1, TAX1BP1, and SQSTM1.^13^, ^14^ Mitophagy is a “double‐edged sword” in tumorigenesis and tumor development depending on the types and stages of tumor. On the one hand, mitophagy is considered as a tumor suppressor, as the inhibition of mitophagy leads to the metabolic reprogramming that mediates malignant transformation, including inducing EMT. On the other hand, mitophagy serves to maintain mitochondrial function to relieve TME stress, which is crucial for cell survival to promote tumor metastasis.^15^, ^16^, ^17^

ICC refers to a highly invasive hepatobiliary neoplasm arising in the intrahepatic biliary tree. Although considered a “rare” cancer in most countries, the incidence of ICC has increased in the recent decades which is responsible for 20% of liver‐related deaths.^18^ Moreover, patients are frequently diagnosed at a locally advanced or metastatic stage with a poor 5‐year survival rates ranging from 25 to 40% even after resection due to the lack of identifiable factors and clinical symptoms.^19^ Unfortunately, the success of available chemotherapeutic regimens or few novel targeted agents is negligible, including the first‐line therapy (gemcitabine and cisplatin). Therefore, there is an urgent necessity to understand the precise mechanism underlying ICC progression to identify novel therapeutic strategies. Accordingly, we assessed the expression of p62 in tumors and adjacent tissues of ICC. We further conducted in vivo and in vitro experiments to demonstrate that p62 was essential for EMT of ICC cells and p62 knockdown might impair EMT and mitochondrial function resulting in the inhibition of ICC growth and progression.

## 2 METHODS

### 2.1 Patients and specimens

Tumor samples were obtained from 156 patients who underwent curative hepatectomy and were pathologically diagnosed with ICC between January 2013 and December 2016 at Liver Cancer Institute, Zhongshan Hospital, Shanghai, China. The ICC cases enrolled met the following criteria (1): pathological diagnosis of ICC; (2) no antitumor treatment before the surgery; (3) no other concurrent malignancies; (4) disease‐free survival time longer than 3 months after hepatectomy; and (5) complete medical records and follow‐up data. Of these, formalin‐fixed paraffin‐embedded specimens of 140 patients with ICC were used for tissue microarray (TMA) construction, and snap‐frozen samples of 16 patients were collected for analyzing p62 expression between tumor and adjacent normal tissues. This study was approved by the ethics committee of Zhongshan Hospital and informed consent was obtained from each patient.

### 2.2 Cell culture and transfection

Human cholangiocarcinoma cell lines HuCCT1, RBE, QBC939, and HCCC‐9810 were purchased from the Cell Bank of Type Culture Collection of Chinese Academy of Sciences and cultured in the RPMI‐1640 medium (HyClone) supplemented with 10% fetal bovine serum (Gibco). QBC939 and HCCC‐9810 cells were transfected with lentivirus vectors encoding short hairpin RNA (shRNA) targeting p62 (shp62) or negative control vectors (shCtrl) (Genechem) in the light of the manufacturer's directions, and stable cell lines with p62 knockdown were selected with 4ug/mL puromycin incubation for 14 days. The shp62 coding sequences were 5’‐CGTCAATAGCAACTGCTCCAA‐3′. The efficiency of p62 knockdown was validated by western blotting analysis.

### 2.3 Western blotting analysis

The total protein of whole cells or tissues were extracted using RIPA cell lysis buffer containing protease inhibitors (Beyotime). After determining protein concentrations using bicinchoninic acid (BCA) protein assay kit (Beyotime), equal amounts of proteins (20ug) were separated by SDS‐PAGE and then transferred onto PVDF membranes. Following blocking in 5% (w/v) non‐fat milk, membranes were incubated with diluent primary and secondary antibodies. BeyoECL kit (Beyotime) was used for visualizing the protein bands. The following antibodies were utilized: anti‐GAPDH (Abcam), anti‐SQSTM1 (Abcam), anti‐E‐cadherin (Cell Signaling Technology), anti‐N‐cadherin (Cell Signaling Technology), anti‐vimentin (Abcam), anti‐Snail (Abcam), anti‐Tomm20 (Abcam), and anti‐COXIV (Abcam).

### 2.4 Quantitative real‐time reverse transcription polymerase chain reaction (qRT‐PCR)

Total RNA was extracted using Trizol reagent (Invitrogen), and RNA was converted to cDNA using cDNA synthesis kit (YEASEN). qRT‐PCR was performed using the SYBR Green Master MIX Kit (Tsingke) according to the manufacturer's instructions. Relative gene expression was evaluated by the 2^−ΔΔCt^ method with GAPDH as an endogenous control. The primer sequences for RT‐PCR are detailed as follows: GAPDH: forward: 5’‐CATGGCCTTCCGTGTTCCTA‐3′, reverse: 5’‐GCGGCACGTCAGATCCA‐3′; p62: forward: 5’‐GACTACGACTTGTGTAGCGTC‐3′, reverse:5’‐AGTGTCCGTGTTTCACCTTCC‐3′; E‐cadherin: forward: 5’‐GTAGGAAGGCACAGCCTGTC‐3′, reverse: 5’‐CAGCAAGAGCAGCAGAATCA‐3′; vimentin: forward: 5’‐CTGCAGGACTCGGTGGACTT‐3′, reverse: 5’‐GAAGCGGTCATTCAGCTCCT‐3’.

### 2.5 Cell migration and invasion assays

To assess cell migration and invasion properties, the chamber was pre‐coated with or without Matrigel (BD Biosciences). The cells (8 × 10^4^cells/well) were seeded into the upper chamber (8 μm pore size) with serum‐free medium in each group, and medium with 10% FBS was added to the lower chamber. After incubation for 24–48 h, the migratory/invasive cells adhering to the lower surface were fixed with 4% paraformaldehyde and stained with crystal violet. The number of cells in the membrane were counted in 10 randomly selected visual fields under a microscope.

### 2.6 Wound healing assay

For wound healing assays, stable transfected and control cells were cultured in 6‐well plates and were grown until reaching confluence. A sterile pipette was used to scratch a wound in the center of the cell monolayers. Images of the wounds were captured using a microscope at 0 and 24 h. The migration rate was calculated as follows: healed migrated cell surface area/total wound area ×100%.

### 2.7 Immunofluorescence cell staining

Cells transfected with lentivirus‐driven shp62 or shCtrl were seeded on sterile coverslips in a 12‐well plate. After 24 h, the cells were divided into two groups based on whether they were treated with 1 mM iron chelator deferiprone (DFP). Then, the cells were fixed with 4% paraformaldehyde and permeabilized with 0.1% Triton X‐100. Subsequently, the cells were incubated with primary and fluorescent secondary antibodies after blocking with 10% goat serum for 1 hour. The immunofluorescence signal was observed using Olympus FV‐3000 confocal microscope (Olympus). The primary antibodies used were as follows: Anti‐TOMM20 (1:500, Abcam); anti‐LC3B (1:50, Abcam); anti‐SQSTM1 (1:800, Abcam); anti‐E‐cadherin (1:200, Cell Signaling Technology); anti‐N‐cadherin (1:200, Cell Signaling Technology); anti‐vimentin (1:200, Abcam). The secondary antibodies including Alexa 488‐conjugated goat anti‐rabbit/mouse IgG and Alexa 594‐conjugated goat anti‐rabbit/mouse IgG from Abcam were used.

### 2.8 Assessment of mitochondrial respiration function

Oxygen consumption rates (OCR) were determined by a Seahorse Extracellular Flux analyzer (Agilent) using the Mito Stress Test Kit (Agilent). Briefly, 5 × 10^3^ viable shp62 cells and their control cells were cultured in the XFe cell culture microplates overnight. Then the cell culture medium was replaced with DMEM XF base medium supplemented with 1 mM pyruvate, 2 mM glutamine, and 10 mM glucose and kept at 37°C for an additional 1 hour without CO_2_. OCR was measured prior to and after sequential injection of 1.5 μM oligomycin, 0.5 μM Carbonyl cyanide‐4(trifluoromethoxy)phenylhydrazone (FCCP), and 0.5 μM of rotenone plus antimycin A. All mitochondrial respiratory parameters were calculated and analyzed according to the manufacturer's instructions and protocols.

### 2.9 Tissue microarray and immunohistochemistry staining

TMA was constructed by Shanghai TUFEI Biotech Co. Ltd. (Shanghai, China). The anti‐SQSTM1, anti‐E‐cadherin, anti‐vimentin, and anti‐Snail antibodies were applied as primary antibodies in immunohistochemistry (IHC) based on a two‐step protocol as previously described.^20^ The staining intensity was categorized into levels 0, 1, 2, and 3. The percentage of positive staining cells was scored as 0 (0%), 1(1–25%), 2 (26–50%), 3 (51–75%), and 4 (76–100%). The product of intensity and percentage score was used as a final score.

### 2.10 Animal xenograft models

All animal experiments were approved by Ethics Committee of Zhongshan Hospital, Fudan University. Four‐week‐old BALB/c nude male mice were purchased from SLAC Laboratory Animal Co. Ltd. (shanghai, China) and fed in specific‐pathogen‐free (SPF) condition. To evaluate the proliferation ability, 5 × 10^6^ QBC939 cells stably transfected with lentivirus‐driven shp62 or shCtrl were injected subcutaneously into the right flanks of nude mice in each group (*n* = 5). Tumor volumes were measured with an external caliper and calculated using the formula π/6 × length × width^2^ every 5 days as we previously described.^20^ Forty days after subcutaneous injection, the mice were sacrificed by cervical dislocation. And serial sections were made for every block from the lung, then metastases were confirmed by HE staining of serial sections.

### 2.11 Statistical analysis

Data are expressed as mean value ± standard deviation (SD). Statistical significance for each variable was estimated by unpaired two‐tailed Student's *t*‐test, Mann–Whitney *U* test, or one‐way ANOVA test using GraphPad Prism 8.0, as appropriate. The correlation between p62 expression and EMT markers' expression were analyzed using Pearson's correlation method. The Pearson correlation coefficient (*r*) were calculated using GraphPad Prism. *P* < 0.05 was considered to indicate a statistically significant difference.

## 3 RESULTS

### 3.1 p62 expression was elevated in ICC tissues and associated with lymph node metastasis and poor prognosis of patients

**FIGURE 1:**
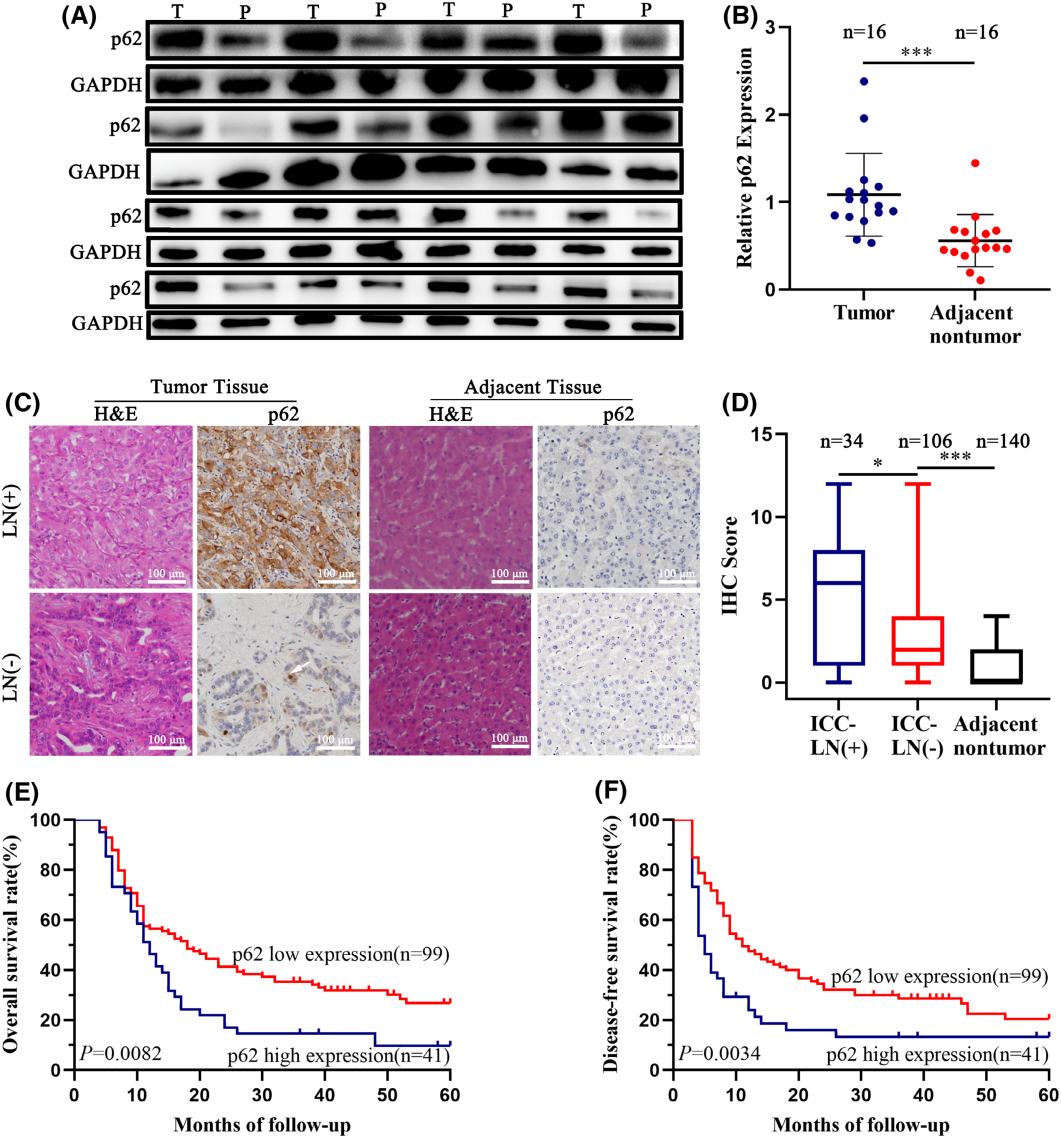
Expression of p62 in tissues of ICC with or without lymph node (LN) metastasis and its effect on prognosis. (A) Western blotting images of p62 expression in 16 paired ICC and adjacent tissues. (B) Statistical analysis of p62 expression in 16 paired ICC and adjacent tissues. (C) Representative H&E and immunohistochemistry staining (IHC) images of p62 expression in ICC and adjacent tissues from 140 patients. Scale bar = 100 μm. (D) Quantitative comparison of IHC scores between ICC and adjacent tissues with [LN (+), *n* = 34] or without LN metastasis [LN (−), *n* = 106]. (E, F) Kaplan–Meier showed overall survival time and disease‐free survival time of 140 patients with different p62 expression. The data are presented as mean ± SD or median (range). Statistical significance was assessed using Student's *t*‐test, log‐rank test, or Mann–Whitney *U* test, as appropriate. *p < 0.05, **p < 0.01, ***p < 0.001.

To identify the potential role of p62 in ICC, we compared the protein expression of p62 in 16 ICC tissue samples with adjacent nontumor tissues (Figure 1A). A significant increase in p62 expression was observed in the tumor tissues compared with the adjacent tissues (*P* < 0.001). The expression in 50% of these tumor samples increased more than twofolds (Figure 1B). The clinical parameters of all the 16 patients are summarized in Table S1–S2.

We further validated our hypothesis using TMA to evaluate the correlation between p62 and prognosis in 140 ICC patients who underwent curative liver resection. Notably, the p62 protein level in cytoplasm was higher in tumor tissues, especially in ICC with lymph node metastasis (Figure 1C). From the data quantified, only nine para‐tumor samples had an IHC score of 3 or 4 points, while most of the tumor tissues had a score of more than 3 points, especially in ICC with lymph node metastasis (Figure 1D). Then, we divided the whole study cohort into p62 high expression (the IHC score >4 points; *n* = 41) and p62 low expression (the IHC score ≤ 4 points; *n* = 99) subgroups, and analyzed the relationship between the level of p62 expression and the clinicopathological characteristics of ICC patients. The results indicated that older patients had higher expression of p62. However, no significant correlation was found between p62 expression and clinical characteristics such as gender, liver cirrhosis, tumor size, tumor number, α‐fetoprotein level, and tumor capsule (Table S2). Moreover, high expression of p62 was significantly associated with lymph node metastasis (*p* = 0.0346) (Figure 1D). The heterogeneity of the degree of p62 expression in different status of lymph node metastasis encouraged us to examine whether p62 expression was associated with the survival of patients with ICC. Kaplan–Meier survival analysis revealed that ICC patients with high levels of p62 had a significantly shorter overall survival and higher cumulative recurrence rate compared with those with low expression of p62 (*p* = 0.0082 and *p* = 0.0034, respectively) (Figure 1E,F).

These observations indicated that high p62 expression in ICC not only contributed to lymph node metastasis, but also influenced patient survival after liver resection.

### 3.2 Inhibition of p62 impaired the metastasis potential of ICC cells in vitro

**FIGURE 2:**
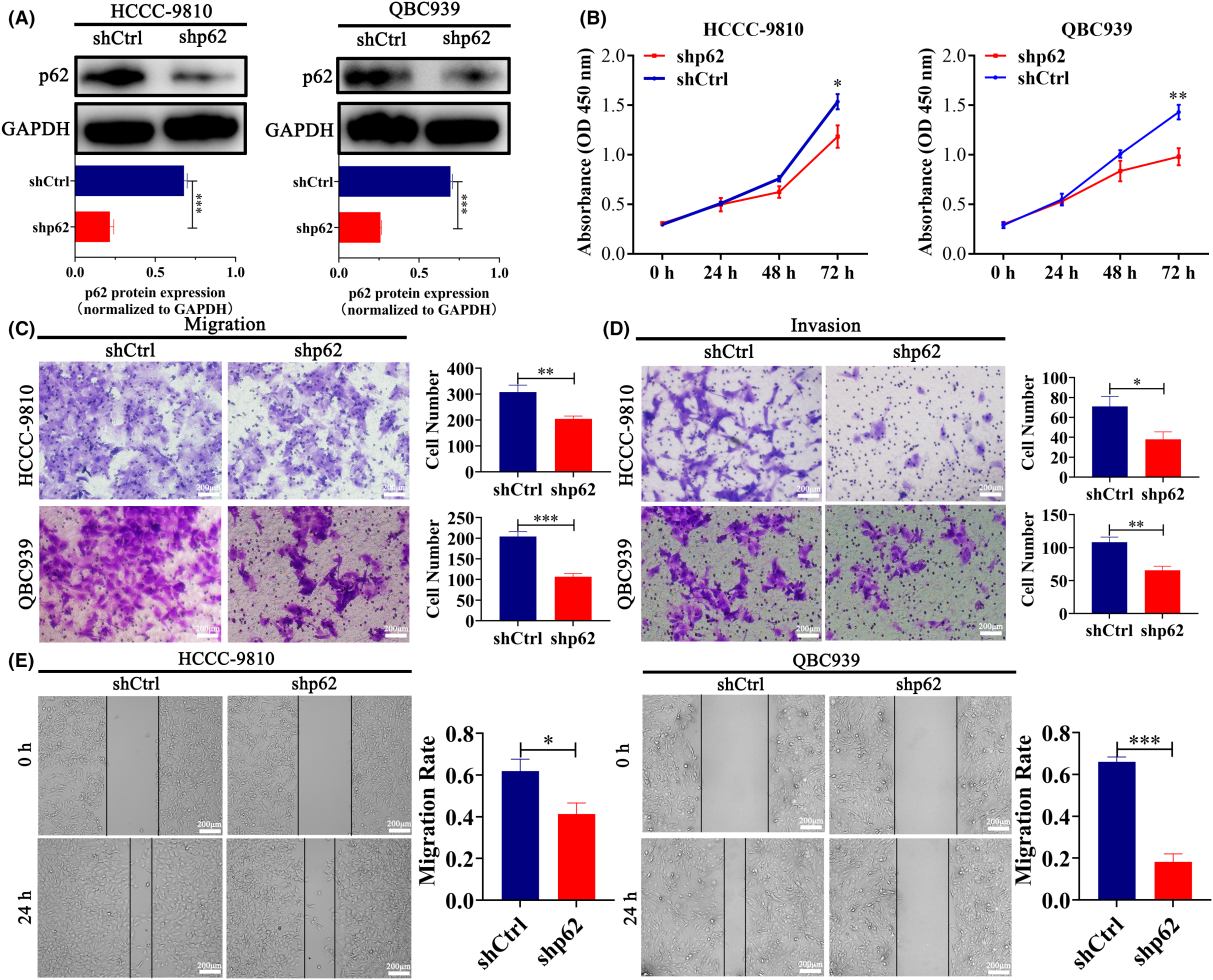
Inhibition of p62 impairs ICC progression in vitro. (A) Western blotting confirmation of p62 knockdown using shRNA in HCCC‐9810 and QBC939 cells. The intensities of bands normalized to GAPDH are quantified in the lower panels. (B) Quantification of cell proliferation of p62 knockdown (shp62) and control (shCtrl) HCCC‐9810 and QBC939 cells by Cell Counting Kit‐8 assay (CCK8). (C‐D) Representative images and quantification of Transwell migration assay and Matrigel invasion assay of shp62 and shCtrl ICC cells. Scale bar = 200 μm (E) Representative images of wound‐healing assays, and histogram analysis of cell migration distance is showed in the right panels. Scale bar = 200 μm. The data are presented as mean ± SD. Statistical significance was assessed using Student's *t*‐test. *p<0.05, **p<0.01, ***p<0.001.

We compared the p62 protein level in HCCC‐9810, HuCCT1, RBE, and QBC939 cells, and observed high p62 expression in HCCC‐9810 and QBC939 cells (Figure S1). To further evaluate the cellular functions, especially metastatic potential, of p62 in vitro, we successfully constructed these two ICC cells with the stable knockdown of p62 by shRNA. The inhibitory efficiency was evaluated by western blotting analysis (Figure 2A). We investigated the role of p62 expression in cell proliferation and found that the inhibition of p62 expression in ICC cells resulted in the decreased proliferation rate (Figure 2B). Cellular migration is a characteristic of metastatic tumors and the first step of invasion. To assess whether p62 downregulation in ICC cells affected cell migration, we performed Transwell migration and wound‐healing assays. The microscopic observation after 24 h revealed a significant delay in the wound closure of p62‐knockdown ICC cells compared with the control cells (Figure 2E). As shown in Figure 2C, silencing p62 expression was associated with a 33.8% and 47.9% reduction in the number of migrated cells through the Transwell chamber in the HCCC‐9810‐shp62 and QBC939‐shp62 cells compared with control cells, respectively. In addition, the Matrigel invasion assay was used to validate the invasive potentials of ICC cell lines. The cells with p62 knockdown showed a significant reduction in their ability to migrate through Matrigel compared with control cells (Figure 2D). Collectively, these in vitro assays suggested that knockdown of p62 expression attenuated the proliferation, migration, and invasion potentials of ICC cells.

### 3.3 p62 inhibition impaired tumor growth and metastasis in vivo

**FIGURE 3:**
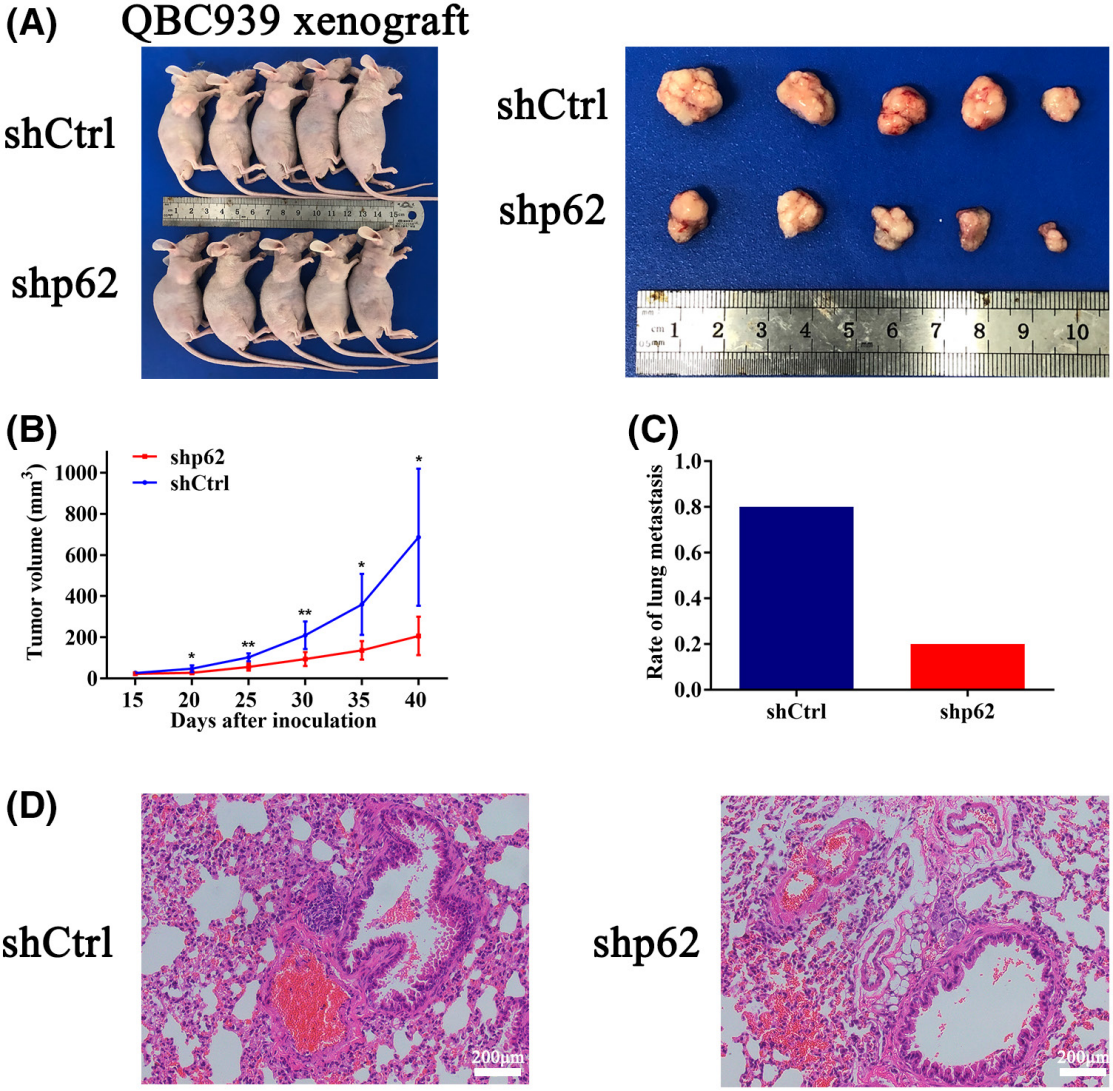
p62 promotes ICC growth and metastasis in vivo. (A) Overview of tumors in BALB/c mice subcutaneously implanted with QBC939‐shp62 or QBC939‐shCtrl cells (*n* = 5/group). (B) Comparison of tumor volumes in mice model implanted with QBC939‐shp62 or QBC939‐shCtrl cells. (C) Rate of lung metastasis in different groups. (D) Representative H&E staining images of lung metastasis. Scale bar = 200 μm. The data are presented as mean ± SD. Statistical significance was assessed using Student's *t*‐test. *p<0.05, **p<0.01.

Next, we constructed a subcutaneous xenograft model using QBC939‐shp62 and QBC939‐shCtrl cells. Two weeks after inoculation, all mice successfully formed palpable tumors. Compared with controls, the mice injected with QBC939‐shCtrl cells showed significantly faster tumor growth which was reflected by increased tumor size (Figure 3A). After inoculation for 40 days, the tumor volume of QBC939‐shCtrl xenografts was 686.4 ± 333.2 mm^3^, which was significantly larger than that derived from QBC939‐shp62 group (207.3 ± 93.6 mm^3^, *p* = 0.0147, Figure 3B). Furthermore, the metastatic analysis in vivo found that the downregulation of p62 reduced lung metastasis capacity in QBC939‐shp62 group mice. In contrast, the lung metastasis rate was 80% (4/5) in the QBC939‐shCtrl group with more metastatic lung nodules (Figure 3C,D). These results indicated that the reduction of p62 expression could significantly inhibit tumor growth and progression of ICC in vivo.

### 3.4 p62 promoted the tumor progression of ICC cells through induction of EMT

**FIGURE 4:**
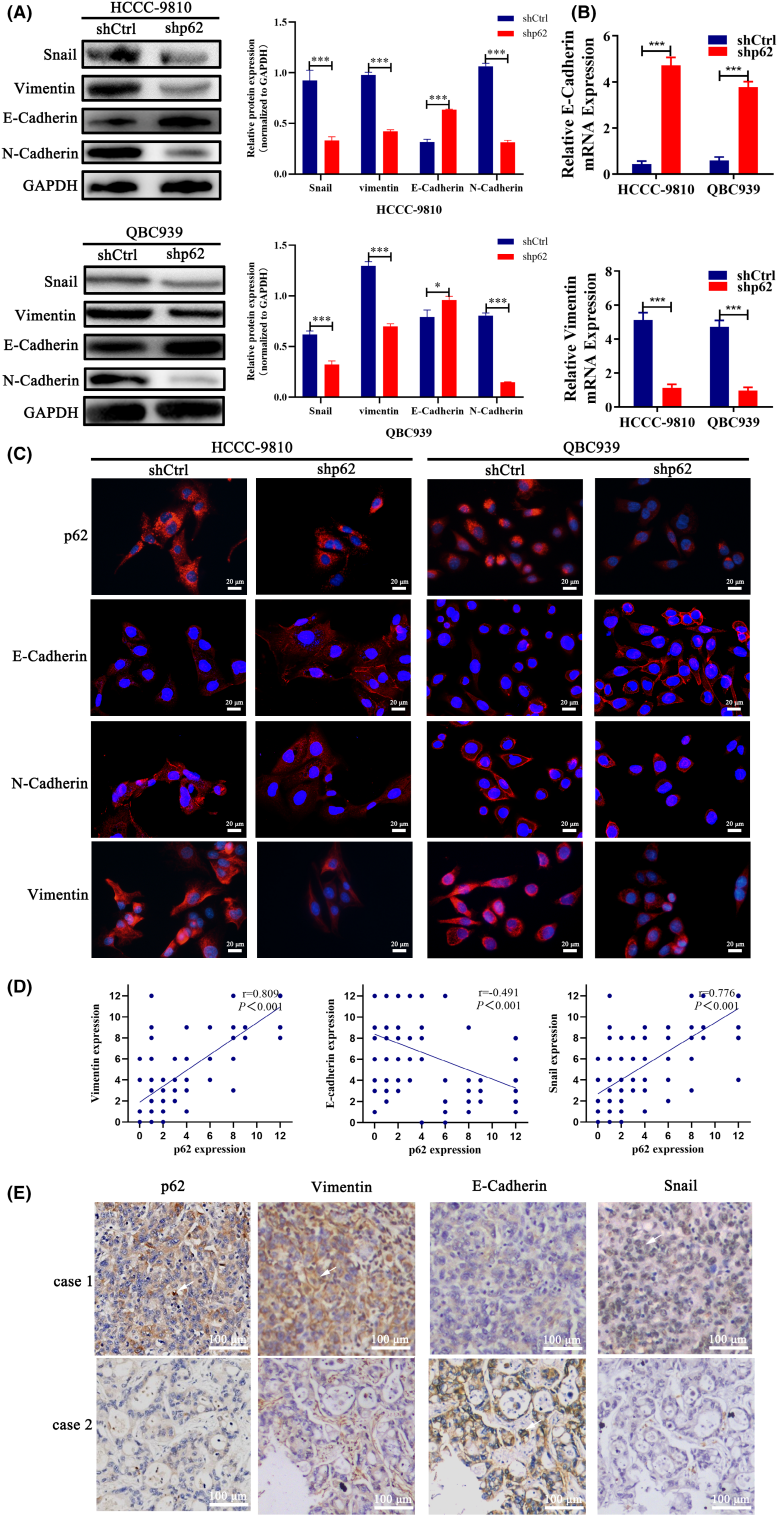
High level of p62 promoted tumor progression through induction of EMT. (A) Western blotting determined N‐cadherin, E‐cadherin, vimentin, Snail proteins expression in p62 knockdown(shp62), and control (shCtrl) HCCC‐9810 and QBC939 cells. The intensities of bands normalized to GAPDH are quantified in the right panels. (B) qRT‐PCR analysis of E‐cadherin and vimentin mRNA levels in ICC cells with different p62 expression. (C) Immunofluorescent staining for p62, E‐cadherin, N‐cadherin, and vimentin of ICC cells with different p62 expression. DAPI stain was used to identify nuclei. Scale bar = 20 μm (D) Correlation between expression of p62 and EMT markers based on IHC scores. (E) Representative images of levels of p62, E‐cadherin, vimentin, and Snail in ICC tissues detected by immunohistochemistry staining. Scale bar = 100 μm The data are presented as mean ± SD. Statistical significance was assessed using Student's *t* test. *p<0.05, **p<0.01, ***p<0.001.

The tumor cells mainly undergo EMT to acquire the migratory and invasive properties. Therefore, we performed western blotting and immunofluorescence assays to determine the expression of EMT‐related markers in ICC cells with different level of p62. The western blotting assay demonstrated a diminished expression of N‐cadherin, Snail and vimentin, and increased expression levels of E‐cadherin in ICC cells after the interference of p62 expression (Figure 4A). The qRT‐PCR results also further confirmed that E‐cadherin mRNA was upregulated in ICC cells with p62 knockdown whereas vimentin mRNA expression was significantly inhibited (Figure 4B). Consistently, the immunofluorescence assay also showed that downregulation of p62 in ICC cells resulted in the increased expression of epithelial marker E‐cadherin and the decreased expression of N‐cadherin and mesenchymal marker vimentin (Figure 4C). We further used the IHC to investigate the relationship between the expression of p62 and EMT‐related markers in the 140 ICC tissues. Based on the IHC scores, p62 expression negatively correlated with E‐cadherin expression (*r* = −0.491, *p* < 0.001), while it was positively correlated to vimentin and Snail expression (*r* = 0.809, *p* < 0.001; *r* = 0.776, *p* < 0.001, Figure 4D). Representative pictures are presented in Figure 4E. Taken together, these data implicated that the higher expression of p62 might significantly promote ICC tumor progression by inducing the EMT process.

### 3.5 p62 inhibition compromised EMT via the regulation of mitochondrial function and mitophagy in ICC cells

**FIGURE 5:**
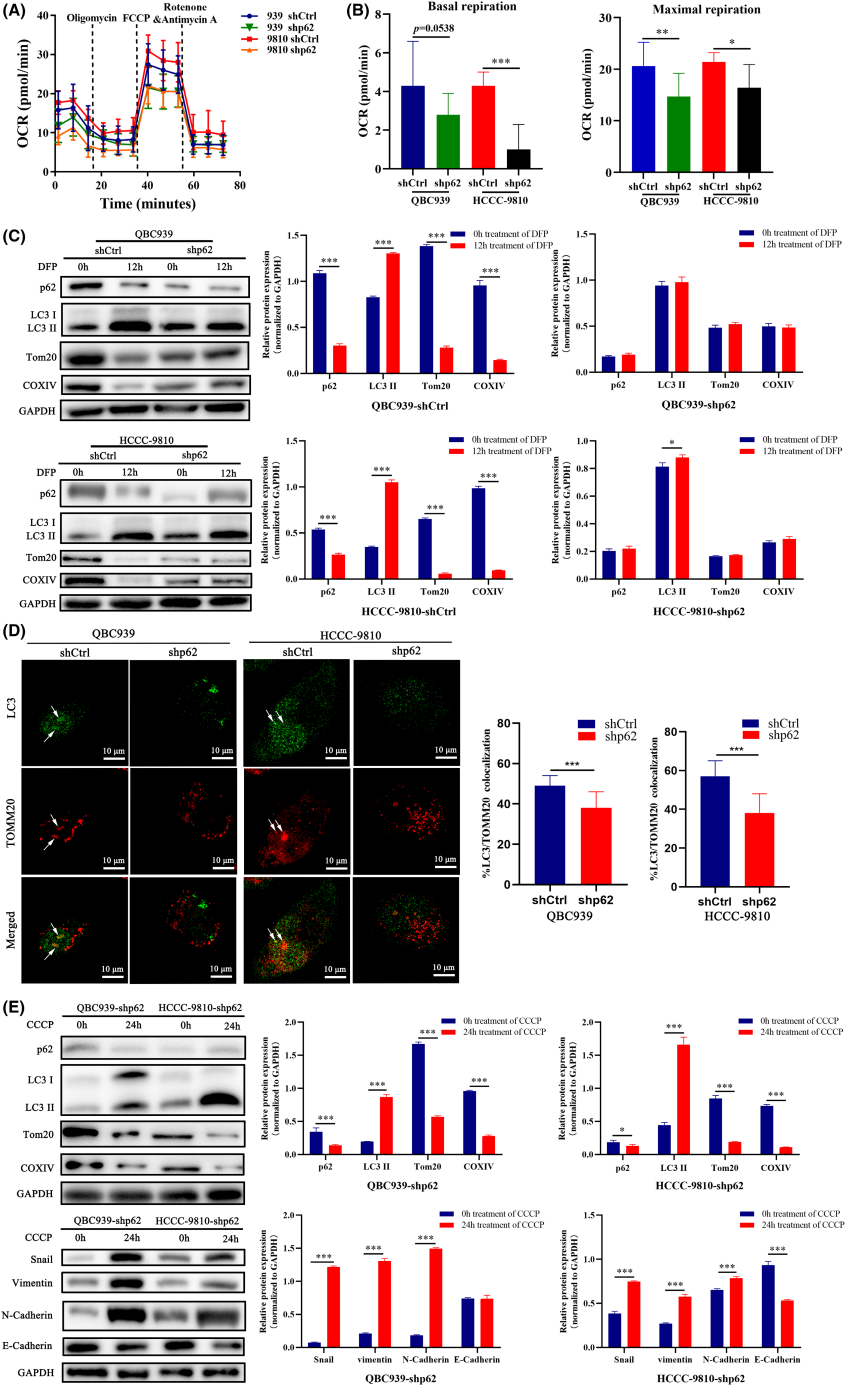
p62 inhibition compromises EMT via regulation of mitochondrial function and mitophagy in ICC cells. (A) Mitochondrial respiration of HCCC‐9810‐shp62 and QBC939‐shp62 or HCCC‐9810‐shCtrl, and QBC939‐shCtrl cells was determined by measuring the oxygen consumption rate (OCR). (B) The rates of basal respiration, maximal respiration was compared between shp62 and shCtrl groups of HCCC‐9810 and QBC939 cells. (C) p62, LC3, TOMM20, and COXIV were assessed by western blotting in shCtrl and shp62 ICC cells after 12 h treatment with the 1 mM iron chelator deferiprone (DFP). The intensities of bands normalized to GAPDH are quantified in the right panels. (D) Immunofluorescent staining for TOMM20 and LC3 of ICC cells with different p62 expression after 1 mM DFP treatment. Arrows indicate colocalization of both markers. Colocalization of TOMM20 and LC3 was analyzed by image J. (E) HCCC‐9810‐shp62 and QBC939‐shp62 cells were treated with 15 μM carbonyl cyanide m‐chlorophnyl hydrazine (CCCP) for 24 h. Immunoblotting assays for p62, LC3, TOMM20, and COXIV, as well as EMT markers (Snail, vimentin, N‐cadherin, and E‐cadherin) were performed. The intensities of bands normalized to GAPDH are quantified in the right panels. The data are presented as mean ± SD. Statistical significance was assessed using Student's *t*‐test. *p<0.05, **p<0.01, ***p<0.001.

The mutual interplay between mitochondrial dysfunction and EMT in tumors has been recently highlighted, which is believed to be associated with progression to a metastatic and drug‐resistant phenotype.^11^ We investigated the functionality of mitochondria in ICC cells after the inhibition of p62 expression. Measuring the mitochondrial OCR which quantified mitochondrial function more precisely demonstrated that the basal and maximal respiratory capacities were significantly reduced in the p62‐knockdown ICC cells compared with the control cells (Figure 5A,B).

As is showed in the results above, the inhibition of p62 caused mitochondrial dysfunction. We hypothesized that p62 might be required for maintenance of mitochondrial respiration through mitophagy which could degrade dysfunctional mitochondria. We then used the iron chelator deferiprone (DFP) which triggered PINK1/Parkin‐independent mitophagy. As shown in Figure 5C, 1 mM DFP treatment induced a significant increase in the expression of LC3, whereas the expression of two mitochondrial proteins, COXIV, and TOMM20, decreased significantly in shCtrl cells. However, DFP had no effect on p62‐knockdown ICC cells as the expression of LC3 and mitochondrial proteins remained unchanged. Meanwhile, we assessed the early stage of mitophagy by visualizing colocalization of mitochondrial marker TOMM20 with the autophagosomal marker LC3. The p62‐knockdown ICC cells were found to have significant reduction of LC3 recruitment to mitochondria after DFP treatment (Figure 5D). To further explore the effect of mitophagy on EMT, we used carbonyl cyanide m‐chlorophnyl hydrazine (CCCP) to induce classic PINK1/Parkin‐dependent mitophagy in shp62 cells which was shown to have decreased level of PINK1/Parkin‐independent mitophagy. Western blotting analysis showed that CCCP treatment restored mitophagy in shp62 cells because of the increase of LC3‐II turnover and the decrease of TOMM20 and COXIV expression. Moreover, induction of mitophagy mediated the increase in the levels of N‐cadherin, Snail, and vimentin proteins, while E‐cadherin expression was decreased (Figure 5E). Together, these results suggested that inhibition of p62 impaired autophagic clearance of dysfunctional mitochondria, and disrupted mitochondrial homeostasis which might be associated with compromised EMT potential of ICC cells.

## 4 DISCUSSION

Mounting evidence has shown that the selective autophagy receptor p62 is a multifunctional protein involved in multiple signaling pathways that regulate inflammation, cancer, and other common diseases. And p62 expression is upregulated in a variety of tumors, such as HCC, pancreatic cancer and breast cancer, but few studies reported this upregulation in ICC.^4^, ^5^, ^6^, ^21^ p62 accumulation is involved in the arsenite‐induced transformation of human hepatic epithelial (L‐02) cells.^22^ Likewise, our results showed there was higher p62 expression in ICC tissues compared with their normal tissues, suggesting that p62 might be involved in the formation of ICC. Moreover, p62 is not only overexpressed in early‐stage of tumor, but also accumulated to promote tumor development.^2^, ^3^, ^22^, ^23^, ^24^ A previous study has reported that p62 could stabilize oncogenic transcription factor TWIST1 which contributed tumor cell proliferation and migration.^25^ Consistent with this, our study found that p62 expression was notably higher in ICC tissues with lymph node metastasis. Intriguingly, recent findings revealed p62 was a double‐edged sword in cancer. For instance, p62 was essential for H2A ubiquitination through inhibiting E3 ligase RNF168s activity, which consequently impaired DNA repair and increased the sensitivity of cancer cells to radiation.^26^ In accordance, Flightless‐I could hinder p62‐dependent selective autophagy, which was associated with accumulation of damage of protein and DNA leading to tumorigenesis in breast cancer.^27^ From a general perspective, these controversial roles of p62 might be derived from its cell‐type dependent role of interactive hub in multiple signaling pathways.

In order to validate the positive association between p62 and tumor progression in ICC, we further revealed that knockdown of p62 inhibited proliferation, migratory, and invasive capabilities of ICC cells in vivo and tumor growth and metastasis in vitro. In the last decades, how cancer cells metastasize from the primary sites have gained a lot of attention, one of which was a reversible dynamic process known as EMT. EMT endows epithelial cancer cells with enhanced motility and invasiveness, thus we conducted a series of experiments to validate the role of p62 in the EMT of ICC. Our data confirmed that p62 downregulation remarkably decreased the expression of mesenchymal marker vimentin, and increased epithelial marker E‐cadherin expression. Additionally, as a key transcription factor of EMT, Snail was also inhibited by p62 knockdown in ICC cell lines and tissues. Consistently, it has been reported that p62 upregulated Snail through the activation of nuclear factor kappa‐light‐chain‐enhancer of activated B cells (NF‐κB) to promote EMT.^28^ In addition to this, as a selective autophagy receptor, p62 could promote EMT of glioblastoma cells through targeting GSK‐3β for degradation.^29^ Given the ability of p62 to promote EMT of ICC, the pharmacological targeting of p62 might represent a feasible strategy with therapeutic potential.

Metabolic plasticity provides cancer cells the ability to cope with the changes of TME. Mitochondria, a major source of ATP provision, are described as a vital player in tumor initiation and progression by reprogramming the bioenergetic cell metabolism.^30^ The interplay between tumor progression and mitochondrial metabolism has been highlighted in the last decades. It has been widely observed that mitochondrial dysfunction was associated with the increasing invasive, metastatic potentials of cancer.^31^ However, the precise mechanisms underlying how dysregulation of mitochondrial metabolism affects tumor progression are still a matter of debate. For this reason, we explored the effect of loss of p62 on mitochondrial metabolism and found that downregulation of p62 impaired mitochondrial function significantly in ICC cells.

EMT is strongly interconnected with autophagy because both play a vital role in the occurrence and development of cancer and are regulated by various same signaling pathways, such as PI3K/AKT/mTOR, Beclin‐1, and JAK/STAT signaling pathways.^32^ Mitophagy is a special type of autophagy that mediates the clearance of dysfunctional or damaged mitochondria to alleviate cellular stress and maintain normal cell growth. It is indicated that mitophagy may play dichotomous roles in tumorigenesis and tumor progression.^16^, ^17^ To mark mitochondria for mitophagy, ubiquitinated mitochondria need receptor proteins to integrate with autophagosomes, such as p62.^14^ Meanwhile, besides PINK1/Parkin‐dependent mitophagy, p62 can promote the ubiquitination of mitochondrial independently of PINK1 and PRKN, and iron chelators can induce this kind of mitophagy.^33^, ^34^ Consequently, we further verified whether p62 protein could regulate mitochondrial function by participating in mitophagy. Our data demonstrated that downregulation of p62 impaired mitochondrial function and DFP‐induced PINK1/Parkin‐independent mitophagy simultaneously by utilizing mitochondrial respiration function test, immunofluorescence assay, and western blotting analysis. We presumed that mitophagy might be involved in mitochondrial regulation, thus facilitating the migration and invasion of ICC cells. Similarly, a previous study revealed that loss of p62 impaired murine myeloid leukemia progression through significantly delaying the removal of damaged mitochondria and impairing mitochondrial respiration.^24^ Nonetheless, in the initiation of carcinogenesis, Parkin‐deficient mice were susceptible to spontaneous hepatocellular carcinoma which indicated that loss of mitophagy‐induced mitochondrial dysfunction and stimulated carcinogenesis.^35^ Meanwhile, a growing number of articles have illustrated that Parkin or mitophagy played a suppressive role in cancer.^36^ These controversies might be ascribed to the fact that mitophagy regulated EMT in multiple ways depending on tumor stage, TME, and metabolic reprogramming.

Overall, the current study revealed that p62 might be involved in the maintenance of mitophagy to keep the balance of mitochondrial dynamics, and thus participate in the metabolic reprogramming and EMT to promote ICC tumor progression. However, specific signaling pathways that p62 are involved in mitotic phagocytosis and mitochondrial dynamics to promote EMT in ICC remains to be further studied. Based on these observations, our study identified that targeting p62 as a potential clinical strategy for the treatment of ICC. In addition, the study also shows that the future drug development of ICC may benefit from pharmacologically targeting mitophagy.

## 5 CONCLUSIONS

In conclusion, we believe that p62 in ICC could promote EMT and tumor progression, which might be caused by the maintenance of mitochondrial function and mitophagy that degrades dysfunctional mitochondria. Our results provide a novel insight that p62 is a potential prognostic and therapeutic target in ICC.

## AUTHOR CONTRIBUTIONS

ZBD, XTF, and JFC conceived and designed the study. YHS, ZT provided research methods. JFC, ZG, XGL, WRL, XZ, AH, XML, QG, GYD, and KS participated in implementation of the study. JFC, ZG, and XGL performed the statistical analysis. JFC, JZ, JF, XTF, and ZBD drafted and revised the manuscript. All authors read and approved the final manuscript.

## FUNDING INFORMATION

This work was supported by the National Natural Science Foundation of China (No. 81972229, 82172610) and Natural Science Foundation of Shanghai (No. 20ZR1473100).

## CONFLICT OF INTEREST

The authors declare that they have no competing interests.

## ETHICS APPROVED AND CONSENT TO PARTICIPATE

This study was approved by the Ethics Committee of Zhongshan Hospital of Fudan University (registration number Y2019‐271). All patients provided their written informed consent for data collection for research purposes.

## CONSENT FOR PUBLICATION

Not applicable.

## Supporting information


Figure S1

Figure S2

Table S1–S2

## Data Availability

The datasets generated and analysed during the current study are available from the corresponding author on reasonable request.
