# Supplementary figures and images for: SQSTM1/p62 in intrahepatic cholangiocarcinoma promotes tumor progression via epithelial–mesenchymal transition and mitochondrial function maintenance
Source: Cancer Med. 2022 Jun 8;12(1):459–71. doi: 10.1002/cam4.4908 (PMC9844629; doi:10.1002/cam4.4908)

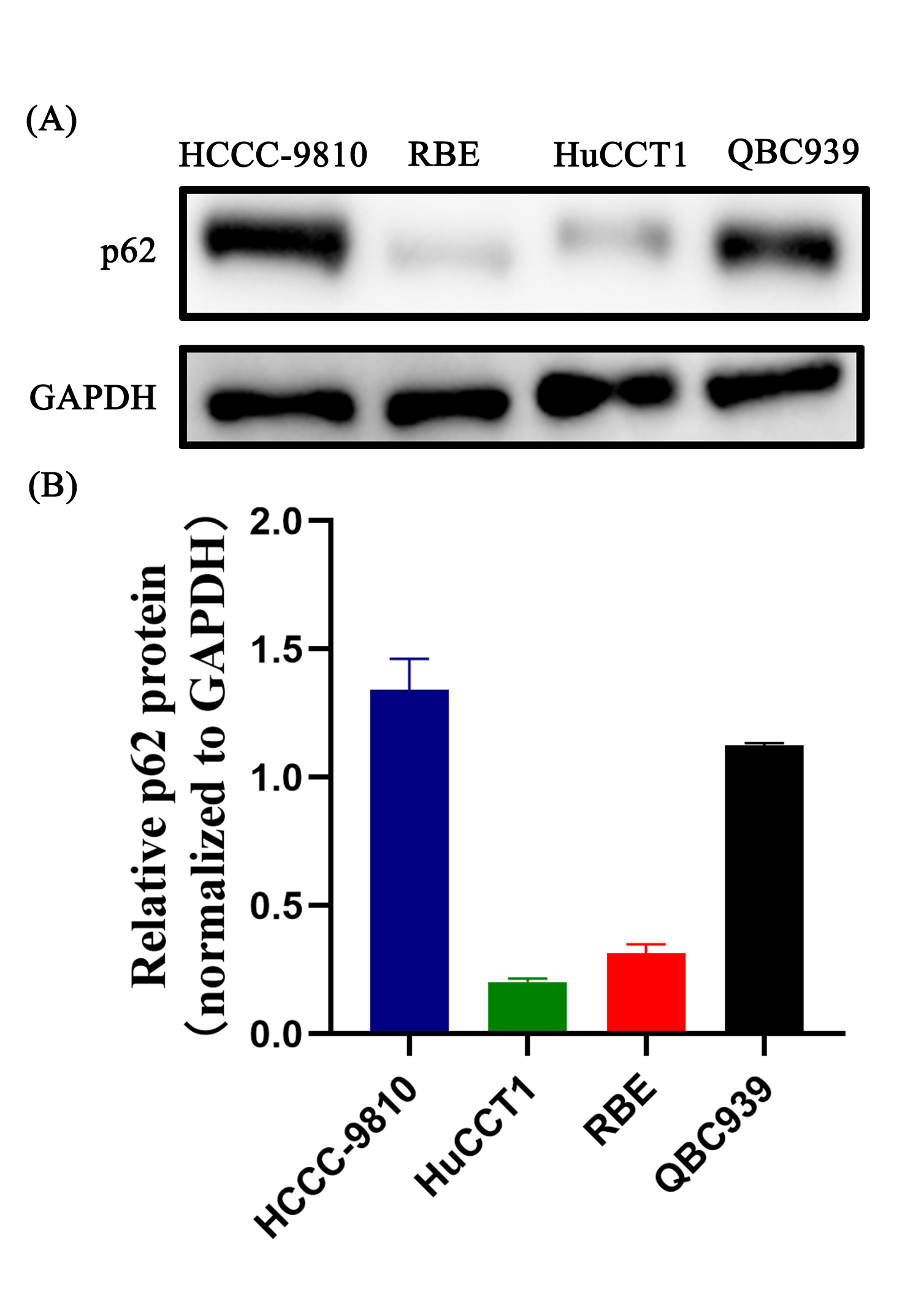

Supplement: Supplementary file 1 — Figure S1 [file CAM4-12-459-s001.tif]

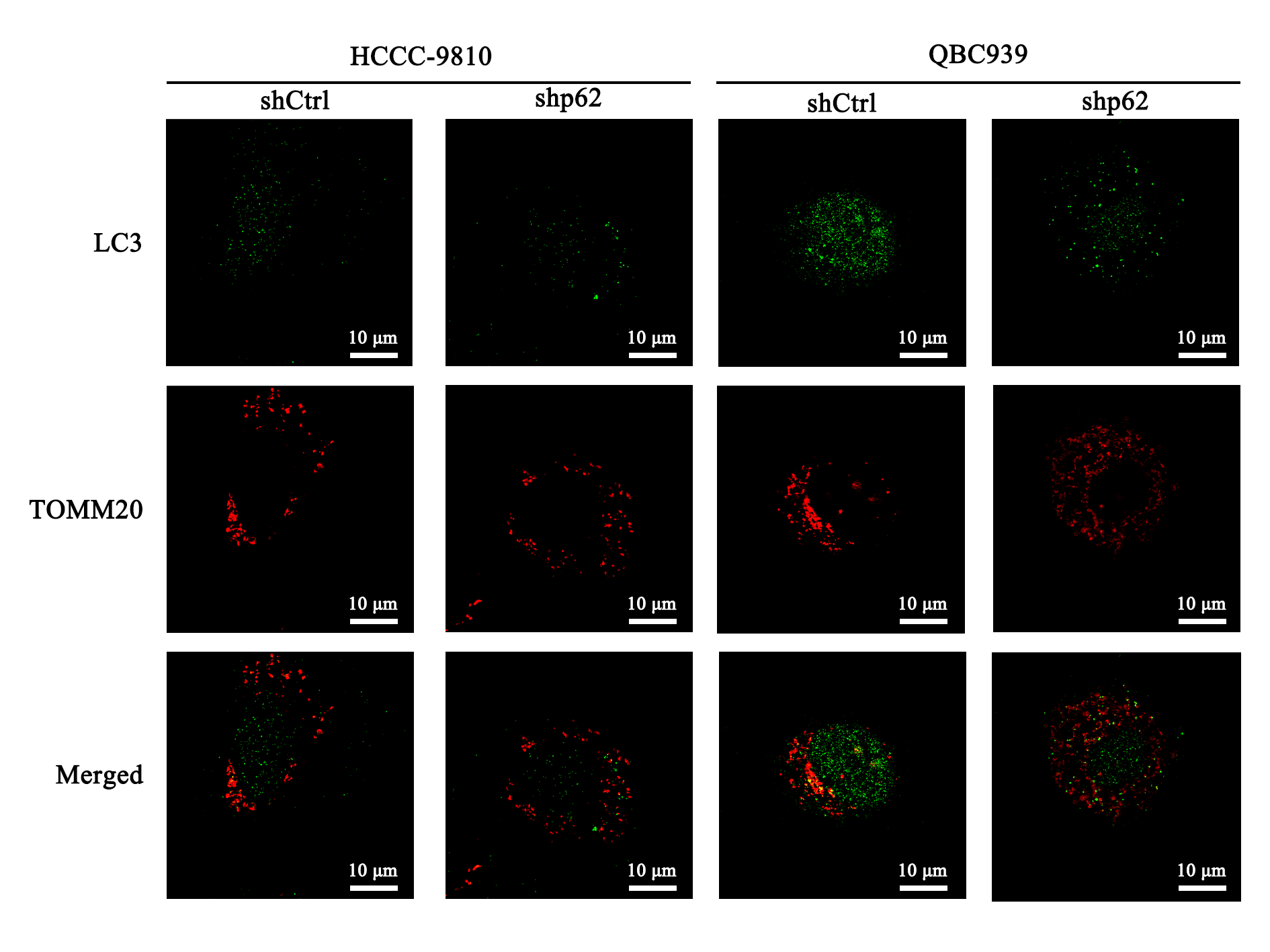

Supplement: Supplementary file 2 — Figure S2 [file CAM4-12-459-s003.tif]
