# Supplementary material for: SQSTM1/p62 in intrahepatic cholangiocarcinoma promotes tumor progression via epithelial–mesenchymal transition and mitochondrial function maintenance
Source: Cancer Med. 2022 Jun 8;12(1):459–71. doi: 10.1002/cam4.4908 (PMC9844629; doi:10.1002/cam4.4908)
Supplement: Supplementary file 3 — Table S1–S2 [file CAM4-12-459-s002.docx]

Supplemental Table 1 Patient baseline characteristics and tumor characteristics of 16 patients

|  | Age  (years) | Gender | HBV (P/N) | AFP (ng/mL) | Child-Pugh grade | Liver cirrhosis (P/N) | Number of tumors | Size of  Tumors | Lymph node metastasis (P/N) |
| --- | --- | --- | --- | --- | --- | --- | --- | --- | --- |
| Case1 | 50 | F | N | 4.7 | A | N | 2 | 9.5 | P |
| Case2 | 66 | M | P | 3.8 | A | P | 3 | 4 | P |
| Case3 | 72 | F | N | 2.2 | A | N | 1 | 7.5 | N |
| Case4 | 47 | M | P | 10.9 | A | N | 2 | 6 | N |
| Case5 | 63 | M | P | 1.7 | A | P | 1 | 2.1 | N |
| Case6 | 60 | M | N | 3.3 | A | N | 2 | 3.5 | N |
| Case7 | 71 | F | N | 1.1 | A | P | 1 | 8 | N |
| Case8 | 65 | M | N | 4.8 | A | N | 1 | 5 | N |
| Case9 | 79 | M | N | 3.9 | A | N | 1 | 12 | N |
| Case10 | 78 | F | P | 1.8 | A | N | 1 | 8 | N |
| Case11 | 65 | F | N | 30.8 | A | N | 1 | 11 | P |
| Case12 | 63 | F | N | 4.6 | A | N | 2 | 2.5 | N |
| Case13 | 60 | M | N | 29.1 | A | N | 1 | 5 | P |
| Case14 | 58 | M | N | 3.4 | A | N | 1 | 5 | N |
| Case15 | 60 | M | N | 2.4 | A | N | 1 | 4.5 | N |
| Case16 | 63 | F | P | 26.4 | A | P | 1 | 3 | N |

M, male; F, female; P, positive; N, negative

Supplemental Table 2 Patient baseline characteristics and tumor characteristics of TMA

| Characteristics | P62 high expression (n=41) | p62 low expression (n=99) | *P* value |
| --- | --- | --- | --- |
| Age (years) |  |  |  |
| ≥55 | 26 | 44 | 0.041 |
| ＜55 | 15 | 55 |  |
| Gender (M/F) | 22/19 | 37/62 | 0.076 |
| Number of tumors |  |  |  |
| Multiple | 5 | 6 | 0.220 |
| Single | 36 | 93 |  |
| Size of tumors (cm) |  |  |  |
| ＜5 | 11 | 20 | 0.390 |
| ≥ 5 | 30 | 79 |  |
| HBV (P/N) | 30/11 | 57/42 | 0.083 |
| Child-Pugh grade (A/B) | 38/3 | 96/3 | 0.358 |
| Liver cirrhosis (P/N) | 13/28 | 44/55 | 0.163 |
| AFP (ng/mL) |  |  |  |
| ＜20 | 37 | 85 | 0.481 |
| ≥20 | 4 | 14 |  |
| Tumor capsule |  |  |  |
| None & Partial | 35 | 82 | 0.712 |
| Complete | 6 | 17 |  |

M, male; F, female; P, positive; N, negative

Statistical significance was assessed using Mann-Whitney *U* test.
